# *catena*-Poly[[diiodidomercury(II)]-μ_2_-2-amino­pyrazine-κ^2^ *N* ^1^:*N* ^4^]

**DOI:** 10.1107/S1600536812006149

**Published:** 2012-02-17

**Authors:** Sadif A. Shirvan, Mohammad R. Asghariganjeh, Manouchehr Aghajeri, Sara Haydari Dezfuli, Farzaneh Hossini

**Affiliations:** a Department of Chemistry, Omidieh Branch, Islamic Azad University, Omidieh, Iran; b Department of Chemistry, Mahshahr Branch, Islamic Azad University, Mahshar, Iran

## Abstract

In the crystal of the title polymeric compound, [HgI_2_(C_4_H_5_N_3_)]_*n*_, the Hg^II^ cation is located on a twofold rotation axis and is coordinated by two I^−^ anions and two 2-amino­pyrazine ligands in a distorted HgI_2_N_2_ tetra­hedral geometry. In the crystal, the 2-amino­pyrazine ligand is equally disordered over two positions about an inversion center, and bridges the Hg^II^ cations with pyrazine N atoms to form a polymeric chain running along the *c* axis. In the polymeric chain, the amino groups link to the coordinated I^−^ anions *via* inter­molecular N—H⋯I hydrogen bonds.

## Related literature

For related structures, see: Sun *et al.* (2009 ▶); Pagola *et al.* (2008 ▶); Boonmak *et al.* (2010 ▶); Gao & Ng (2011 ▶); Goher *et al.* (2008 ▶).
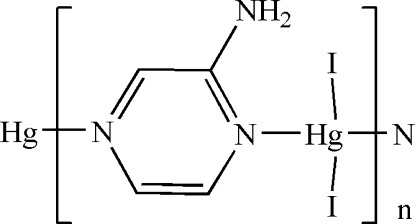


## Experimental

### 

#### Crystal data

[HgI_2_(C_4_H_5_N_3_)]
*M* *_r_* = 549.50
Monoclinic, 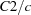

*a* = 15.3389 (19) Å
*b* = 6.8791 (8) Å
*c* = 9.6239 (11) Å
β = 103.828 (10)°
*V* = 986.1 (2) Å^3^
*Z* = 4
Mo *K*α radiation
μ = 21.81 mm^−1^
*T* = 298 K
0.50 × 0.05 × 0.04 mm

#### Data collection

Bruker APEXII CCD area-detector diffractometer
Absorption correction: multi-scan (*SADABS*; Bruker, 2001 ▶) *T*_min_ = 0.275, *T*_max_ = 0.417
2648 measured reflections
933 independent reflections
911 reflections with *I* > 2σ(*I*)
*R*_int_ = 0.173

#### Refinement

*R*[*F* ^2^ > 2σ(*F* ^2^)] = 0.083
*wR*(*F* ^2^) = 0.224
*S* = 1.08
933 reflections
52 parameters
H-atom parameters constrained
Δρ_max_ = 2.67 e Å^−3^
Δρ_min_ = −2.69 e Å^−3^

### 

Data collection: *APEX2* (Bruker, 2007 ▶); cell refinement: *SAINT* (Bruker, 2007 ▶); data reduction: *SAINT*; program(s) used to solve structure: *SHELXTL* (Sheldrick, 2008 ▶); program(s) used to refine structure: *SHELXTL*; molecular graphics: *ORTEP-3 for Windows* (Farrugia, 1997 ▶); software used to prepare material for publication: *WinGX* (Farrugia, 1999 ▶).

## Supplementary Material

Crystal structure: contains datablock(s) I, global. DOI: 10.1107/S1600536812006149/xu5467sup1.cif

Structure factors: contains datablock(s) I. DOI: 10.1107/S1600536812006149/xu5467Isup2.hkl

Additional supplementary materials:  crystallographic information; 3D view; checkCIF report

## Figures and Tables

**Table 1:** Selected bond lengths (Å)

Hg1—I1	2.6373 (13)
Hg1—N1^i^	2.497 (11)

Symmetry code: (i) 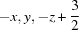.

**Table 2:** Hydrogen-bond geometry (Å, °)

*D*—H⋯*A*	*D*—H	H⋯*A*	*D*⋯*A*	*D*—H⋯*A*
N2—H2*A*⋯I1^i^	0.86	2.83	3.67 (3)	169

Symmetry code: (i) 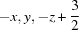.

**Fig. 1.**
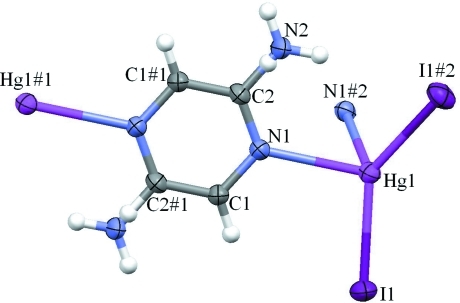
The molecular structure of the title molecule, with the atom-numbering scheme. Displacement ellipsoids are drawn at the 30% probability level. [Symmetry codes: #1: -x, 1 - y, 1 - z; #2: -x, y, 3/2 - z].

**Fig. 2.**
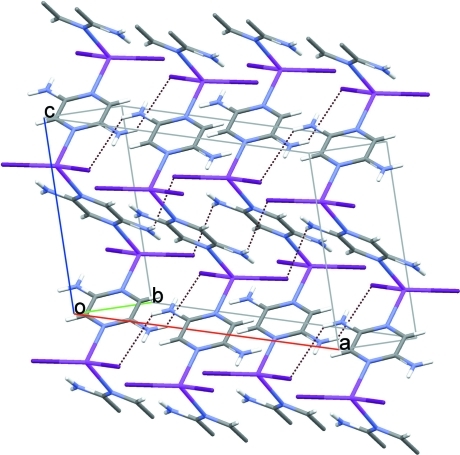
A packing diagram of the title complex. Hydrogen bonds are shown as dashed lines.

## supplementary crystallographic information

### Comment

2-Aminopyrazine is a good ligand and numerous complexes with 2-aminopyrazine have been prepared, such as that of silver (Sun *et al.*, 2009), copper (Pagola *et al.*, 2008), cobalt, iron and cadmium (Boonmak *et al.*, 2010) and zinc (Gao & Ng, 2011; Goher *et al.*, 2008). Here, we report the synthesis and structure of the title compound.

The asymmetric unit of the title compound, (Fig. 1), contains one half molecule. The Hg^II^ atom is four-coordinated in a distorted tetrahedral configuration by two N atoms from two 2-aminopyrazine and two I atoms. The Hgd-I and Hg—N bond lengths angles are collected in Table 1.

Intermolecular N—H···I hydrogen bonds (Table 2) seem to be effective in the stabilization of the polymeric structure (Fig. 2).

### Experimental

For the preparation of the title compound, a solution of 2-aminopyrazine (0.15 g, 1.50 mmol) in methanol (10 ml) was added to a solution of HgI_2_ (0.55 g, 1.50 mmol) in methanol (10 ml) and the resulting colorless solution was stirred for 15 min at room temperature. This solution was left to evaporate slowly at room temperature. After one week, colorless needle crystals of the title compound were isolated (yield 0.63 g, 76.4%).

### Refinement

H atoms were positioned geometrically with C—H = 0.93 and N—H = 0.86 Å, and constrained to ride on their parent atoms with *U*_iso_(H) = 1.2*U*_eq_(C,N). In the crystal, the 2-aminopyrazine ring is equqlly disordered over two positions about an inversion center.

### Crystal data

**Table:**

[HgI_2_(C_4_H_5_N_3_)]	*F*(000) = 944
*M**_r_* = 549.50	*D*_x_ = 3.701 Mg m^−^^3^
Monoclinic, *C*2/*c*	Mo *K*α radiation, λ = 0.71073 Å
Hall symbol: -C 2yc	Cell parameters from 2648 reflections
*a* = 15.3389 (19) Å	θ = 2.7–26.0°
*b* = 6.8791 (8) Å	µ = 21.81 mm^−^^1^
*c* = 9.6239 (11) Å	*T* = 298 K
β = 103.828 (10)°	Needle, colorless
*V* = 986.1 (2) Å^3^	0.50 × 0.05 × 0.04 mm
*Z* = 4	

### Data collection

**Table:**

Bruker APEXII CCD area-detector diffractometer	933 independent reflections
Radiation source: fine-focus sealed tube	911 reflections with *I* > 2σ(*I*)
Graphite monochromator	*R*_int_ = 0.173
ω scans	θ_max_ = 26.0°, θ_min_ = 2.7°
Absorption correction: multi-scan (*SADABS*; Bruker, 2001)	*h* = −18→18
*T*_min_ = 0.275, *T*_max_ = 0.417	*k* = −8→8
2648 measured reflections	*l* = −11→8

### Refinement

**Table:**

Refinement on *F*^2^	Secondary atom site location: difference Fourier map
Least-squares matrix: full	Hydrogen site location: inferred from neighbouring sites
*R*[*F*^2^ > 2σ(*F*^2^)] = 0.083	H-atom parameters constrained
*wR*(*F*^2^) = 0.224	*w* = 1/[σ^2^(*F*_o_^2^) + (0.1405*P*)^2^ + 17.0935*P*] where *P* = (*F*_o_^2^ + 2*F*_c_^2^)/3
*S* = 1.08	(Δ/σ)_max_ = 0.002
933 reflections	Δρ_max_ = 2.67 e Å^−^^3^
52 parameters	Δρ_min_ = −2.69 e Å^−^^3^
0 restraints	Extinction correction: *SHELXTL* (Sheldrick, 2008), Fc^*^=kFc[1+0.001xFc^2^λ^3^/sin(2θ)]^-1/4^
Primary atom site location: structure-invariant direct methods	Extinction coefficient: 0.015 (2)

### Special details

**Table:**

Geometry. All e.s.d.'s (except the e.s.d. in the dihedral angle between two l.s. planes) are estimated using the full covariance matrix. The cell e.s.d.'s are taken into account individually in the estimation of e.s.d.'s in distances, angles and torsion angles; correlations between e.s.d.'s in cell parameters are only used when they are defined by crystal symmetry. An approximate (isotropic) treatment of cell e.s.d.'s is used for estimating e.s.d.'s involving l.s. planes.
Refinement. Refinement of *F*^2^ against ALL reflections. The weighted *R*-factor *wR* and goodness of fit *S* are based on *F*^2^, conventional *R*-factors *R* are based on *F*, with *F* set to zero for negative *F*^2^. The threshold expression of *F*^2^ > σ(*F*^2^) is used only for calculating *R*-factors(gt) *etc*. and is not relevant to the choice of reflections for refinement. *R*-factors based on *F*^2^ are statistically about twice as large as those based on *F*, and *R*- factors based on ALL data will be even larger.

### Fractional atomic coordinates and isotropic or equivalent isotropic displacement parameters (Å^2^)

**Table:**

	*x*	*y*	*z*	*U*_iso_*/*U*_eq_	Occ. (<1)
C1	0.0725 (10)	0.383 (2)	0.5415 (17)	0.042 (3)	
H1	0.1234	0.3063	0.5710	0.050*	
C2	−0.0746 (11)	0.455 (2)	0.542 (2)	0.044 (3)	
H2C	−0.1274	0.4240	0.5694	0.054*	0.50
N1	−0.0046 (8)	0.3371 (17)	0.5809 (12)	0.040 (2)	
N2	−0.154 (2)	0.409 (5)	0.578 (4)	0.059 (8)	0.50
H2A	−0.1586	0.3048	0.6251	0.071*	0.50
H2B	−0.1999	0.4856	0.5531	0.071*	0.50
Hg1	0.0000	0.05992 (11)	0.7500	0.0451 (6)	
I1	0.16288 (9)	−0.07523 (19)	0.76493 (17)	0.0629 (7)	

### Atomic displacement parameters (Å^2^)

**Table:**

	*U*^11^	*U*^22^	*U*^33^	*U*^12^	*U*^13^	*U*^23^
C1	0.038 (7)	0.040 (7)	0.051 (7)	0.005 (6)	0.016 (6)	0.008 (7)
C2	0.034 (7)	0.046 (8)	0.055 (9)	−0.003 (5)	0.016 (6)	0.005 (6)
N1	0.039 (6)	0.035 (5)	0.050 (6)	0.002 (4)	0.018 (5)	0.004 (5)
N2	0.054 (17)	0.062 (17)	0.070 (17)	0.020 (14)	0.032 (15)	0.034 (14)
Hg1	0.0390 (8)	0.0411 (8)	0.0576 (8)	0.000	0.0164 (5)	0.000
I1	0.0484 (10)	0.0655 (10)	0.0793 (11)	0.0199 (5)	0.0241 (7)	0.0154 (5)

### Geometric parameters (Å, º)

**Table:**

C1—N1	1.363 (19)	N1—Hg1	2.497 (11)
C1—C2^i^	1.38 (2)	N2—H2A	0.8600
C1—H1	0.9300	N2—H2B	0.8600
C2—N1	1.33 (2)	Hg1—I1^ii^	2.6372 (13)
C2—H2C	0.9300	Hg1—I1	2.6373 (13)
C2—C1^i^	1.38 (2)	Hg1—N1^ii^	2.497 (11)
C2—N2	1.38 (4)		
			
N1—C1—C2^i^	119.6 (14)	C1—N1—Hg1	117.9 (10)
N1—C1—H1	120.2	C2—N2—H2A	120.0
C2^i^—C1—H1	120.2	C2—N2—H2B	120.0
C1^i^—C2—H2C	119.0	H2A—N2—H2B	120.0
N1^i^—C2—H2C	149.0	N1^ii^—Hg1—N1	80.4 (5)
N1—C2—C1^i^	121.6 (15)	N1^ii^—Hg1—I1^ii^	100.6 (3)
N1—C2—N2	120.0 (17)	N1—Hg1—I1^ii^	110.8 (3)
C1^i^—C2—N2	118.2 (18)	N1^ii^—Hg1—I1	110.8 (3)
C2—N1—C1	118.6 (13)	N1—Hg1—I1	100.6 (3)
C2—N1—Hg1	122.9 (10)	I1^ii^—Hg1—I1	138.71 (7)
			
C1^i^—C2—N1—C1	3 (3)	C2—N1—Hg1—N1^ii^	−68.8 (12)
N2—C2—N1—C1	179 (2)	C1—N1—Hg1—N1^ii^	102.6 (12)
C1^i^—C2—N1—Hg1	174.4 (13)	C2—N1—Hg1—I1^ii^	29.0 (13)
N2—C2—N1—Hg1	−10 (3)	C1—N1—Hg1—I1^ii^	−159.7 (10)
C2^i^—C1—N1—C2	−3 (3)	C2—N1—Hg1—I1	−178.4 (12)
C2^i^—C1—N1—Hg1	−174.8 (12)	C1—N1—Hg1—I1	−7.0 (11)

Symmetry codes: (i) −*x*, −*y*+1, −*z*+1; (ii) −*x*, *y*, −*z*+3/2.

### Hydrogen-bond geometry (Å, º)

**Table:**

*D*—H···*A*	*D*—H	H···*A*	*D*···*A*	*D*—H···*A*
N2—H2*A*···I1^ii^	0.86	2.83	3.67 (3)	169

Symmetry code: (ii) −*x*, *y*, −*z*+3/2.
